# Effectiveness of *Sopoongsan* for chronic upper body pruritus in patients with atopic or seborrheic dermatitis: A pilot study protocol for a randomized, double-blind, placebo-controlled, parallel trial

**DOI:** 10.1097/MD.0000000000031470

**Published:** 2022-11-18

**Authors:** Jung-Hee Jang, Purumea Jun, Gunhyuk Park, Ojin Kwon, Yujin Choi, Hye-Sun Lim, Byeong Cheol Moon, Kyuseok Kim

**Affiliations:** a Clinical Medicine Division, Korea Institute of Oriental Medicine, Daejeon, Republic of Korea; b University of Science and Technology (UST), Campus of Korea Institute of Oriental Medicine, Korean Convergence Medical Science major, Daejeon, Republic of Korea; c Herbal Medicine Resources Research Center3, Korea Institute of Oriental Medicine, Jeollanam-do, Republic of Korea; d Department of Ophthalmology, Otolaryngology and Dermatology of Korean Medicine, College of Korean Medicine, Kyung Hee University, Seoul, Republic of Korea.

**Keywords:** cytokines, histamine, protocol, pruritus, *Sopoongsan*

## Abstract

Chronic pruritus persists for > 6 weeks and is known to decrease patients’ quality of life. Due to the complex pathological mechanism of chronic pruritus, there is still a lack of satisfactory therapeutic agents; therefore, complementary therapies are required to improve itching symptoms. In the Republic of Korea, *Sopoongsan*, an herbal formula, has been used to treat itching, dizziness, and skin paralysis. To our knowledge, this is the first study to evaluate whether *Sopoongsan* improves chronic pruritus and to identify *Sopoongsan*-related changes in the immune response in patients with chronic upper body pruritus.

**Methods:**

A randomized, double-blind, placebo-controlled parallel trial will be conducted to assess 20 patients with chronic upper body pruritus for 3 months who have been diagnosed with allergic atopic dermatitis or seborrheic dermatitis. The patients will be randomly allocated to either the placebo-control (n = 10) or treatment (n = 10) group. The total study period will be 8 weeks (i.e., administration of *Sopoongsan* or placebo drugs for 4 wk and follow-up for 4 wk). Participants will be allowed to receive external treatment, except for antipruritic medications administered orally, throughout the study period. The primary outcome measure will be the numeric rating scale results for itching, whereas the secondary outcome measures will be questionnaire survey (Dermatological Life Quality Index and Epworth Sleepiness Scale) findings and the immune response index, including interferon gamma, interleukin-4, immunoglobulin E, thymic stromal lymphopoietic protein, and histamine, to investigate the biological mechanisms underlying chronic pruritus.

**Discussion and Conclusions:**

We expect that the results of this study will provide important clinical evidence regarding the effectiveness of *Sopoongsan* on itching symptoms, quality of life, sleep disturbance, and changes in the immune response. The findings will help elucidate the mechanism underlying the therapeutic effect of *Sopoongsan* for chronic pruritus and lay the foundation for further studies in this area.

## 1. Introduction

Chronic pruritus is described as an irritating sensation that elicits a desire to scratch and persists for more than 6 weeks. Scratching can aggravate dermal conditions, cause emotional disturbance and sleep loss, and decrease the quality of life.^[1,2]^ The cause of pruritus can be dermatological (e.g., atopic eczema and psoriasis), systemic (kidney failure and liver cirrhosis), neuropathic (notalgia paresthetica), or psychogenic. Chronic pruritus is widespread, with a high estimated prevalence of 15%,^[3]^ and is very difficult to treat.^[4]^ There are no approved pharmacological drugs for the treatment of chronic pruritus.^[5]^ Sedating antihistamines are commonly used as first-line pharmacological therapy for pruritus, but often have only modest efficacy in clinical practice.^[1]^ Therefore, complementary treatment strategies that overcome these limitations are required; however, randomized and controlled trial reports on various pharmacological and nonpharmacological treatments for chronic pruritus are scarce.

*Sopoongsan* is a traditional medicine that is widely used in clinical practice to improve skin diseases based on a nationwide study.^[6–9]^ *Sopoongsan*, called Xiao-feng-san in Chinese, has been reported as the most commonly used traditional medicine for dampness and heat-based pruritus in atopic dermatitis and urticaria; it has also been shown to exert anti-inflammatory, antioxidation, and antiallergic effects.^[7–9]^ *Sopoongsan* is divided into two prescriptions, depending on the source. The first prescription is Waikezhengzong, which contains *Saposhnikovia divaricata*, *Atractylodes lancea*, *Schizonepeta tenuifolia*, *Arctium lappa*, *Glycyrrhiza uralensis*, *Rehmannia glutinosa*, *Gypsum fibrosum*, *Clematis armandii*, *Cryptotympana pustulata*, *Linum usitatissimum*, *Sophora flavescens*, *Angelica sinensis*, and *Anemarrhena asphodeloides*, and is highly effective against dampness and heat-based atopic dermatitis, urticaria, and eczema. As a result of administering *Sopoongsan* to patients with chronic urticaria for 28 days in a randomized controlled trial (RCT), an improvement in patients’ itching symptoms and sleep status and a reduction in serum interferon-gamma (IFN-γ) levels were observed. After administering *Sopoongsan* for 8 weeks in patients with atopic dermatitis, the patients showed an improvement in atopic lesion scores, along with an improvement in itching and sleep status.^[10,11]^ The other prescription is composed of *Notopterygium incisum*, *Pericarpium Citri Reticulatae*, *Cryptotympana pustulata*, *Schizonepeta tenuifolia*, *Smilax china*, *Bombyx batryticatus*, *Saposhnikovia divaricata*, *Agastache rugosa O. Ktze.*, *Magnolia officinalis Rehd*, *Glycyrrhiza uralensis*, *Panax ginseng*, and *Ligusticum chuanxiong Hort* from Dong-uibogam in the Republic of Korea. In contrast to the former, it is more commonly used for dry lesions rather than Waikezhengzong *Sopoongsan*, as it strengthens the vital force and improves the circulation of fluid qi. In animal models in which histamine and ear swelling have been induced, *Sopoongsan* has been confirmed to inhibit mast cell degranulation and histamine secretion, reduce ear swelling, and decrease the levels of nuclear factor kappa B, tumor necrosis factor-alpha, interleukin (IL)-8, and IL-6. However, to date, clinical studies on the effectiveness of *Sopoongsan* used in the Republic of Korea for patients with dermatological diseases have been limited.

The biological mechanisms underlying chronic pruritus are complex and multifactorial.^[1]^ The itch-sensitive neurons originating in the skin are stimulated by several pruritogens, including histamine, immune cells, keratinocytes, and nerves.^[4]^ Histamine activates neurons via the histamine receptor 1, leading to the opening of the heat- and capsaicin-gated transient receptor potential (TRP) channel. TRP channels are key transduction ion channels on the plasma membrane that exacerbate the excitation of neurons and promote itching. Moreover, other non-histaminergic endogenous pruritogens are released from keratinocytes, such as cytokine thymic stromal lymphopoietic protein (TSLP), which activates neurons via the TSLP receptor leading to the opening of TRP ankyrin 1 channels. Activating TRP leads to depolarization, action potential firing, and transmission of itch-neuronal signals from the periphery to the central nervous system.^[4]^ The itch signal is transmitted to areas of the brain that are involved in sensation, evaluative processes, emotion, reward, and memory.^[1]^ Collectively, the analysis of pruritus-related immune cytokines may help elucidate the therapeutic biological mechanisms by which *Sopoongsan* can benefit patients with chronic pruritus. Nonetheless, RCTs investigating the *Sopoongsan*-induced changes in the immune response in serum samples of patients with chronic pruritus are limited.

In this study, we report our study design and plans for evaluating the effectiveness of *Sopoongsan* in patients with chronic pruritus and the feasibility for analyzing data collected during the whole process of the trial. Furthermore, we will elucidate the mechanism underlying the effectiveness of *Sopoongsan* in patients with chronic pruritus by measuring the levels of immune-related cytokines. The specific objectives of the study include the verification of research feasibility to assess the effectiveness of *Sopoongsan* for improving skin itching in patients with chronic upper body pruritus and the investigation of the possibility of immune indicators as therapeutic response biomarkers in response to *Sopoongsan* for skin pruritus.

## 2. Methods

### 2.1. Study design and setting

**Figure 1.**
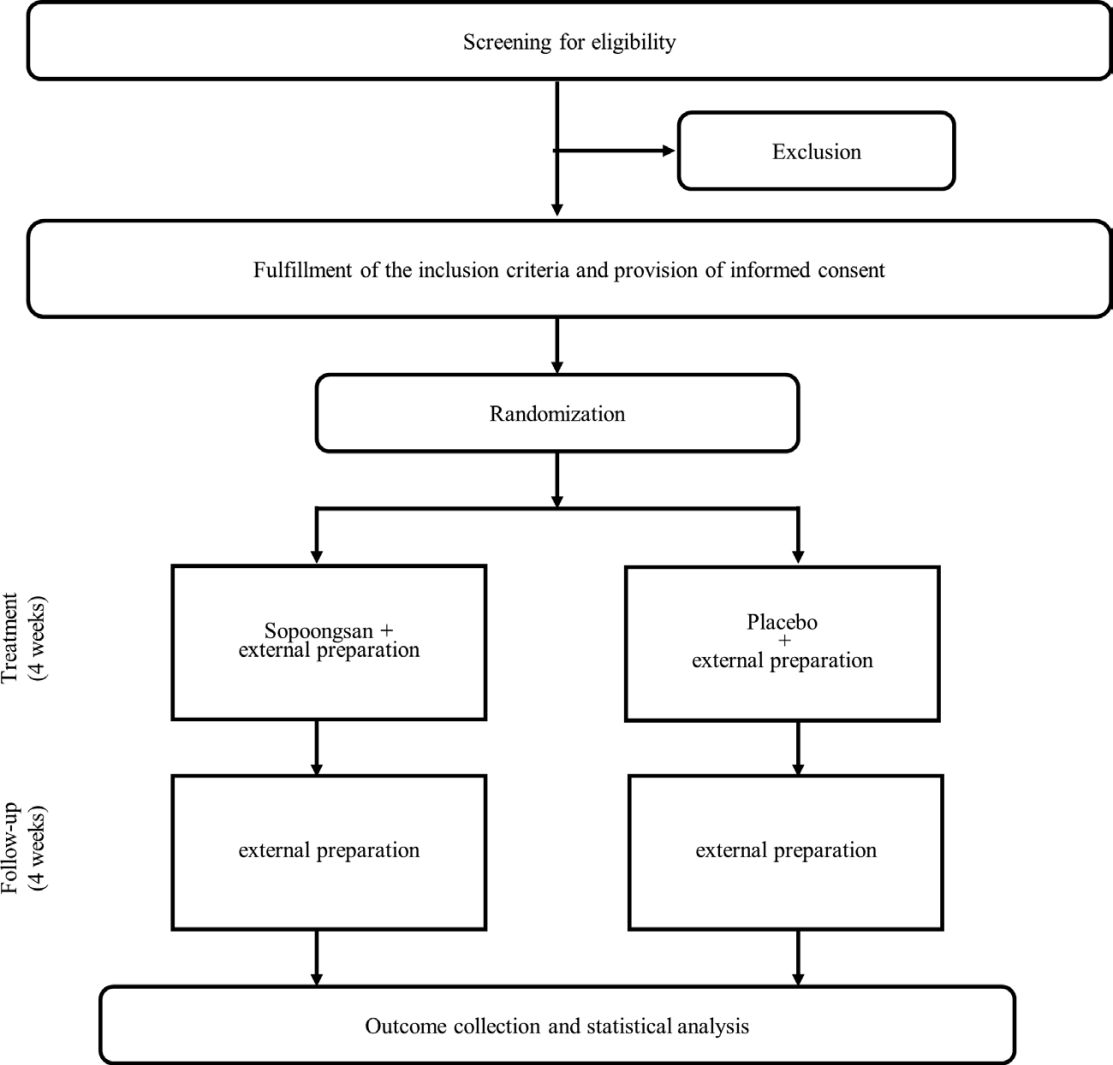
The study design flowchart showing details of the randomized controlled trial.

This clinical study will be a randomized placebo-controlled trial investigating the effectiveness of *Sopoongsan* in the treatment of upper body pruritus in patients with allergic atopic or seborrheic dermatitis. *Sopoongsan* or placebo will be administered orally three times daily for 4 weeks, with a follow-up of 4 weeks. A flow chart describing the clinical study design is illustrated in Figure 1. The effect of *Sopoongsan* on itching will be assessed by improvements in itching symptoms, Dermatology Life Quality Index, presence of sleep disorders due to itching, and changes in immune indicators in the blood. The study setting and site of data collection will be the Clinical Trial Center at the Kyung Hee Korean Medical Hospital, Kyung Hee University, in the Republic of Korea.

### 2.2. Participant recruitment

A total of 20 participants with upper body pruritus will be recruited through posters placed in the hospital and community, local newspapers, and subway advertisements from November 2022 to December 2023. The participants who meet the eligibility criteria (Table 1) will receive information regarding the clinical study during a consultation with the coordinator and provide written informed consent for their participation in this study. Subsequently, all participations will be randomly allocated to either the treatment or control group.

**Table 1:** Patient eligibility criteria.

Inclusion criteria
(1) Patients aged ≥ 19 years (2) NRS score of ≥ 3 for upper body pruritus (3) Pruritus of ≥ 3 months (4) Diagnosis: allergic atopic or seborrheic dermatitis (5) Provided voluntary written informed consent to participate in this clinical study
Exclusion criteria
(1) NRS score of ≥ 7 for upper body pruritus (2) History of taking oral adrenal corticosteroids (steroids) or immunosuppressants for skin itching within the last 4 weeks (3) Skin diseases caused by bacterial or viral infections (4) Clinical abnormality of liver and renal function tests (i.e., ALT, AST, ALP) ≥ 2 times the upper limit of normal or creatinine > 2.0 mg/dL at screening (5) Severe acute cardiovascular disease (i.e., heart failure, myocardial infarction, stroke, hypertension, or diabetes) (6) Serious conditions of hyperlipidemia, anemia, active pulmonary tuberculosis, thyroid disease, and other infectious and systemic diseases (7) Past or present malignant tumors (8) Antipsychotic drug treatment within 2 months before screening test (9) History of acute or chronic pancreatitis or gastrointestinal surgery (except for simple appendix surgery or hernia surgery) (10) Genetic problems such as galactose intolerance, Lapp lactase deficiency, or glucose-galactose malabsorption (11) History of hypersensitivity reactions to the constituents of the clinical trial treatments (12) History of drug or alcohol abuse (13) Pregnant and lactating women or in case or accepting the implementation of an appropriate contraceptive method among women of pregnant potential who are likely to become pregnant (except for women who have undergone sterilization) (14) Plan to participate in other clinical trials after enrollment in this clinical trial, or who have participated in other clinical trials during the 30 days prior to registration in this clinical trial (15) Inappropriate patients as considered by the investigator for other reasons

ALP = alkaline phosphatase, ALT = alanine aminotransferase, AST = aspartate aminotransferase, NRS = numeric rating scale.

### 2.3. Randomization and blinding

The allocation of eligible patients to the control or *Sopoongsan* treatment group at a 1:1 ratio will be performed by an independent statistician using computer-generated block randomization. Each participant will be selected via systematic sampling and receive an identification code enclosed in an opaque envelope. This will be a double-blind study because the participants, researchers, and assessors collecting the data will be blinded to group assignment. If the blinding was disrupted such as in a medical emergency, the principal investigator will hold a researchers’ meeting to decide if unblinding is required. In this case, the unblinding will be informed to the institutional review board (IRB) within 24 h. The enrollment of participants, allocation sequence, and assignment of participants will be the responsibility of the Clinical Trial Center at the Kyung Hee Korean Medical Hospital, Kyung Hee University.

### 2.4. Interventions

All participants in both groups will be allowed to receive external treatment, except for antipruritic medications administered orally and herbal medicine treatment. All participants will orally take either *Sopoongsan* or placebo drugs three times daily, which will be packed in opaque sachets, in granular form. Discontinuation of the intervention by participants may occur because of withdrawal of informed consent by participants or their legal representatives, violation of the eligibility criteria during the clinical trial, occurrence of adverse events, and < 70% compliance with the medication criteria. Medication adherence (number of medicines received/number of medicines to be received) will be assessed by the clinical research coordinator every time the participants visit the clinic.

#### 2.4.1. Treatment group: Sopoongsan.

The treatment group will receive *Sopoongsan*, which contains the following 12 dried medicinal herbs: *Notopterygium incisum*, *Pericarpium Citri Reticulatae*, *Cryptotympana pustulata*, *Schizonepeta tenuifolia*, *Smilax china*, *Bombyx batryticatus*, *Saposhnikovia divaricata*, *Agastache rugosa O. Ktze.*, *Magnolia officinalis Rehd*, *Glycyrrhiza uralensis*, *Panax ginseng*, and *Ligusticum chuanxiong Hort*. The formula will be supplied by the manufacturer of the National Institute for Korean Medicine Development (Republic of Korea) and is manufactured in compliance with Good Manufacturing Practice under the Ministry of Food and Drug Safety guidelines. The final form of *Sopoongsan* is granular.

#### 2.4.2. Control group: placebo.

The control group will be administered a placebo formulation containing lactose, starch, and caramel coloring, which will be manufactured using the same process and by the same manufacturer as those for *Sopoongsan.*

### 2.5. Outcome measures

To evaluate the effect of *Sopoongsan* on skin itching, the intensity of pruritus, as assessed using a numeric rating scale (NRS), will be the primary outcome. The secondary outcome measures will include findings from instruments that evaluate the quality of life, sleep impairment, and changes in the immune response. The schedule for outcome measurements is illustrated in Table 2.

**Table 2:** Schedule for enrollment, clinical outcomes, and safety assessment.

Period	Screening	Treatment			Follow-up*
Visit	Screening	Visit 1	Visit 2	Visit 3	Visit 4
Week	0 week (baseline)	1 week	2 weeks	4 weeks	8 weeks
Provide informed consent	**○**				
Identification of inclusion and exclusion criteria	**○**				
Demographics	**○**				
Medical and disease history	**○**				
Clinical laboratory examination	**○**			**○**	
Vital signs	**○**			**○**	
NRS	**○**	**○**	**○**	**○**	**○**
DLQI		**○**	**○**	**○**	**○**
ESS		**○**	**○**	**○**	**○**
Blood collection for immune analysis	**○**			**○**	
Supply of drug for clinical trial		**○**	**○**		
Medication compliance			**○**	**○**	**○**
Identification of adverse reaction(s)			**○**	**○**	**○**
Identification of concomitant drug change		**○**	**○**	**○**	

DLQI = Dermatological Life Quality Index, ESS = Epworth sleep scale, NRS = numeric rating scale.

* Follow-up by phone interviews.

#### 2.5..1. Primary outcome: intensity of pruritus.

The NRS is a numerical rating scale anchored at 0 and 10 that is marked by patients to indicate their intensity of itch. It has been validated in large-scale studies for chronic pruritus.^[12]^ The intensity of pruritus, as assessed using the NRS, will be the primary outcome. The measurement will be performed at baseline (visit 1), 2 weeks, and 4 weeks during and after treatment (visits 2 and 3) and at follow-up after 4 weeks (visit 4) for both groups.

#### 2.5.2. Secondary outcomes.

##### 2.5.2.1. Quality of life.

Chronic pruritus can greatly reduce the patients’ quality of life. The Dermatological Life Quality Index , an instrument for both pruritus and other dermatological conditions, is widely used and validated.^[12]^ The measurement will be performed at baseline (visit 1), 2 weeks, and 4 weeks during and after treatment (visits 2 and 3) and at follow-up after 4 weeks (visit 4) for both groups.

##### 2.5..2..2. Sleep impairment.

The multifactorial nature of chronic pruritus requires comprehensive diagnostic procedures and lengthy therapies. It is important to treat visible scratch lesions and accompanying disorders, including sleep disorders. The Epworth Sleepiness Scale is a scale developed for determining sleep impairment.^[12]^ The measurement will be performed at baseline (visit 1), 2 weeks, and 4 weeks during and after treatment (visits 2 and 3) and at follow-up after 4 weeks (visit 4) for both groups.

##### 2.5.2.3. Molecular analysis.

Investigating changes in serum cytokine levels may help us understand the mechanism of action of *Sopoongsan* in patients with chronic pruritus. The serum levels of pruritus-related cytokines such as IFN-γ, IL-4, immunoglobulin E (IgE), and TSLP will be analyzed.^3^ The assessment will be conducted at baseline (visit 0) and 4 weeks after treatment (visit 3) for both groups. Blood collected from patients in both groups will be immediately centrifuged at a temperature below 4°C. The obtained plasma and serum samples will be stored at a temperature below −80°C until further analysis. The samples will be analyzed by Global Clinical Central Laboratories (Republic of Korea).

##### 2.5.2.4. Feasibility.

The feasibility of the recruitment process will be measured for conducting a large-scale RCT. The proportion of the number of participants who have been recruited, screened, met the inclusion criteria, agreed to participate, and dropped out will be used to calculate.

### 2.6. Safety assessment and adverse events

A clinical laboratory examination will be performed at baseline, before drug administration, and at 4 weeks after completing drug administrations (visit 3), as a safety assessment for all participants. The liver and renal function can possibly be affected by herbal medicines^[13]^; therefore, liver and renal function tests will be performed additionally, as well as serum potassium concentration assessment because *Glycyrrhiza radix*, a component of *Sopoongsan*, can induce hypokalemia.^[14]^ All adverse events, which are undesirable and/or unexpected medical findings that were absent before commencing this clinical trial, will be recorded. The IRB at Kyung Hee University will be reported, and they will determine whether the trial should be discontinued. The causal link between the adverse events and the procedure will be categorized into six stages. Appropriate treatment and follow-up will be provided for severe adverse events.

### 2.7. Statistical methods

#### 2.7.1. Sample size.

This study will be an exploratory clinical trial to determine the degree of symptomatic improvement that can be achieved with *Sopoongsan* treatment in patients with upper body chronic pruritus. However, statistical hypotheses and effect sizes for the estimation of the sample size cannot be established. Therefore, as a pilot study based on research feasibility, this study considers a total of 20 subjects, 10 per group.

#### 2.7.2. Statistical analysis.

All statistical analyses will be conducted using SAS® version 9.4 (SAS Institute, Inc., Cary, NC). The *p*-values will be two-sided and set at 5% level of significance. A complete set analysis will be conducted to analyze the effectiveness of the intervention, and a per-protocol analysis will be additionally verified. Missing data will be analyzed using the last observation carried forward imputation method. For efficacy analysis, the mixed-effect model for repeated measures will be used, with each group and visit time as fixed factors and subjects as random factors. Student’s paired *t* test will be used to compare the factors before and after treatment. All adverse events (including severe adverse events) will be classified by group.

### 2.8. Data management and monitoring

Information required by the protocol will be collected by the clinical research coordinator by filling out the case report forms. Data coding will be conducted; two research assistants will independently enter the data into Excel sheets, and the results will be verified by cross-checking. All documents obtained from the clinical trial will be preserved in confidential archives at the Kyung Hee Korean Medicine Hospital for 3 years. No information will be released without the permission of the principal investigator. The regular monitoring for patient safety, investigating adverse events, and reviewing quality control of the data will be performed by an independent monitoring committee not related to this study.

### 2.9. Ethical considerations, informed consent, and study registration

The IRB at Kyung Hee University Korean Medicine Hospital approved the protocol version 1.3 (Jun, 16, 2022) of this clinical trial and accepted responsibility for supervising all aspects of the study (KOMCIRB 2022-05-006-002). All participants will be willing to participate in this clinical trial and submit a signed informed consent form. Before consent, all participants and their families will receive an explanation of the potential risks and benefits. The study protocol (version 1.3) was registered with the Clinical Research Information Service of the Republic of Korea (KCT0007537). In the case of significant protocol changes, the research coordinator will request supplementation of the IRB and trial registries.

## 3. Discussion

Pruritus is the most common symptom in dermatology and constitutes more than one-third of dermatological presentations.^[12]^ However, there is limited evidence regarding pharmacological treatment methods for chronic pruritus, and clinical studies investigating the effectiveness and mechanism of action of pharmacological treatments for chronic pruritus are scarce.^[1]^ Thus, the present study aims to investigate the clinical effectiveness of *Sopoongsan* as pharmacological therapy in patients with chronic upper body pruritus.

In the Republic of Korea, the efficacy of *Sopoongsan* is approved for “itching caused by rising wind” by the Ministry of Food and Drug Safety and is specified in Dong-uibogam. To reflect the concept of “rising wind,” we will limit the participants’ lesions to the upper body in patients with allergic atopic or seborrheic dermatitis in the present study. Lesions on the upper part of the human body (i.e., head, neck, and upper limbs) are often caused by wind-warmth and wind-heat because wind tends to move upwards. Therefore, in the present study, based on the indications for *Sopoongsan*, the area corresponding to “rising wind” was limited to the upper body. In addition, in Korean traditional medicine, it is thought that symptoms of dry skin in the upper body are more likely to develop as a result of blood deficiency. Blood deficiency is known as any pathological change characterized by the deficiency of blood, which fails to nourish organs, tissues, and meridians/channels. Blood deficiency induces a pattern/syndrome marked by dry, rough, itchy, shriveled skin with rhagades, withering and loss of hair, numbness of the body surface, contraction of hands and feet, lusterless complexion, pale nails, dizziness and blurred vision, pale tongue, and fine pulse. *Sopoongsan* from Dong-uibogam is widely used to treat dry lesions in the Republic of Korea.

Itching is a response to skin conditions that cause allergic reactions or inflammatory responses. Recently, the understanding of the neuroimmune axis has uncovered new targets for the treatment of chronic pruritus. The skin barrier is functionally divided into three types based on immune cells. Type 1 immunity involves responses mediated by adaptive cells, such as type 1 helper T (Th1) cells and cytotoxic T cells, characterized by the production of INF-γ, and tumor necrosis factor-alpha. The type 1 immune response provides protection against intracellular pathogens and tumor cells and can promote chronic inflammatory processes, such as autoimmune skin diseases and allergic contact dermatitis. Type 2 immunity is mediated by adaptive type 2 helper T (Th2) cells as well as innate immune cells, including basophils, mast cells, and eosinophils, producing the effector type 2 cytokines IL-4, IL-5, and IL-13, which promote allergic inflammation and IgE production. Upstream promoters of the type 2 inflammatory process include TSLP, which can be directly secreted by keratinocytes.^[3]^ The Th1/Th2 immune balance is closely related to various immunological diseases, including allergies. Many investigators have revealed that Th2 immunity is responsible for allergic immune responses and the subsequent pathogenesis of allergic inflammatory diseases. Chronic inflammatory skin diseases (i.e., atopic dermatitis) are known to increase the expression of Th2 cytokines (i.e., IL-4, IL-5, and IL-13) in peripheral blood.^[15]^ As such, Th1/Th2 immune balance can be a very important indicator for itching, and we aim to analyze the changes in cytokines associated with Th1/Th2 immune responses to investigate the mechanism of action of *Sopoongsan* on chronic itching.

The proposed study has the limitation of being a small pilot clinical trial. However, this study could be a basis for large-scale clinical studies in the future because no current evidence clarifies the effectiveness of *Sopoongsan* in the treatment of chronic pruritus and pruritus-related cytokines changes. Another limitation is that targeting patients with dermatological diseases (i.e., atopic dermatitis and seborrheic dermatitis) and chronic pruritus to investigate the effectiveness of *Sopoongsan* is not specifically determined. The efficacy of *Sopoongsan* is on “itching that the wind rises” rather than confining to a disease, which is why the area of the lesions is limited to the upper body. Based on the results of this study, further research is expected.

## 4. Conclusions

This study will be the first clinical trial on *Sopoongsan*, which is used in the Republic of Korea, and will investigate the clinical effectiveness of *Sopoongsan* for the symptoms of upper body pruritus and changes in the immune response in patients with allergic atopic or seborrheic dermatitis. We expect to demonstrate improvements in chronic pruritus, including the intensity of pruritus and patients’ quality of life, as well as changes in the levels of pruritus-related cytokines to obtain an insight into the mechanism underlying the effectiveness of *Sopoongsan* in chronic pruritus and help identify potential alternative therapies for chronic pruritus.

## Author contributions

**Conceptualization:** Jung-Hee Jang, Purumea Jun, Gunhyuk Park, Hye-Sun Lim, Byeong Cheol Moon, Kyuseok Kim.

**Data curation:** Jung-Hee Jang, Purumea Jun, Gunhyuk Park, Ojin Kwon, Yujin Choi, Kyuseok Kim.

**Formal analysis:** Jung-Hee Jang, Purumea Jun, Gunhyuk Park, Ojin Kwon, Yujin Choi.

**Funding acquisition:** Byeong Cheol Moon.

**Investigation:** Kyuseok Kim.

**Methodology:** Jung-Hee Jang, Purumea Jun, Gunhyuk Park, Kyuseok Kim.

**Project administration:** Jung-Hee Jang, Purumea Jun, Gunhyuk Park, Ojin Kwon, Yujin Choi, Hye-Sun Lim, Byeong Cheol Moon.

**Resources:** Jung-Hee Jang, Purumea Jun, Gunhyuk Park, Ojin Kwon, Yujin Choi.

**Software:** Jung-Hee Jang, Purumea Jun, Gunhyuk Park, Ojin Kwon, Yujin Choi.

**Supervision:** Hye-Sun Lim, Byeong Cheol Moon, Kyuseok Kim.

**Validation:** Jung-Hee Jang, Purumea Jun, Hye-Sun Lim, Byeong Cheol Moon, Kyuseok Kim.

**Visualization:** Jung-Hee Jang, Purumea Jun.

**Writing—original draft:** Jung-Hee Jang, Purumea Jun.

**Writing—review and editing:** Gunhyuk Park, Ojin Kwon, Yujin Choi, Hye-Sun Lim, Byeong Cheol Moon, Kyuseok Kim.
